# Neuroinflammation trajectories precede cognitive impairment after experimental meningitis—evidence from an in vivo PET study

**DOI:** 10.1186/s12974-019-1692-0

**Published:** 2020-01-04

**Authors:** Vijayasree V. Giridharan, Allan Collodel, Jaqueline S. Generoso, Giselli Scaini, Rico Wassather, Sudhakar Selvaraj, Rodrigo Hasbun, Felipe Dal-Pizzol, Fabricia Petronilho, Tatiana Barichello

**Affiliations:** 1 Faillace Department of Psychiatry and Behavioral Sciences, McGovern Medical School, The University of Texas Health Science Center at Houston (UTHealth), Houston, TX USA; 2 Experimental Physiopathology Laboratory, Graduate Program in Health Sciences, Graduate Program in Health Sciences, Health Sciences Unit, University of Southern Santa Catarina (UNESC), Criciúma, SC Brazil; 3 Micro Analysis Group, Keyence Corporation of America, Austin, TX USA; 4 Division of Infectious Disease, Department of Medicine, McGovern Medical School, UTHealth, Houston, TX USA; 5 Laboratory of Neurobiology of Inflammatory and Metabolic Processes, Postgraduate Program in Health Sciences, University of South Santa Catarina (UNISUL), Tubarao, SC Brazil

**Keywords:** Meningitis, Post-meningitis, Microglia, TSPO, Inflammation, PET, Cognition

## Abstract

**Background:**

Bacterial meningitis is a devastating central nervous system (CNS) infection with acute and long-term neurological consequences, including cognitive impairment. The aim of this study was to understand the association between activated microglia-induced neuroinflammation and post-meningitis cognitive impairment.

**Method:**

Meningitis was induced in male Wistar rats by injecting *Streptococcus pneumoniae* into the brain through the cisterna magna, and rats were then treated with ceftriaxone. Twenty-four hours and 10 days after meningitis induction, rats were imaged with positron emission tomography (PET) using [^11^C]PBR28, a specific translocator protein (TSPO) radiotracer, to determine in vivo microglial activation. Following imaging, the expression of TSPO, cardiolipin, and cytochrome *c*, inflammatory mediators, oxidative stress markers, and glial activation markers were evaluated in the prefrontal cortex and hippocampus. Ten days after meningitis induction, animals were subjected to behavioral tests, such as the open-field, step-down inhibitory avoidance, and novel object recognition tests.

**Results:**

Both 24-h (acute) and 10-day (long-term) groups of rats demonstrated increased [^11^C]PBR28 uptake and microglial activation in the whole brain compared to levels in the control group. Although free from infection, 10-day group rats exhibited increased expression levels of cytokines and markers of oxidative stress, microglial activation (IBA-1), and astrocyte activation (GFAP) similar to those seen in the 24-h group. Acute meningitis induction also elevated TSPO, cytochrome *c*, and caspase-3 levels with no change in caspase-9 levels. Furthermore, upregulated levels of TSPO, cytochrome *c*, and caspase-3 and caspase-9 were observed in the rat hippocampus 10 days after meningitis induction with a simultaneous reduction in cardiolipin levels. Animals showed a cognitive decline in all tasks compared with the control group, and this impairment may be at least partially mediated by activating a glia-mediated immune response and upregulating TSPO.

**Conclusions:**

TSPO-PET could potentially be used as an imaging biomarker for microglial activation and long-term cognitive impairment post-meningitis. Additionally, this study opens a new avenue for the potential use of TSPO ligands after infection-induced neurological sequelae.

## Introduction

Meningitis is a devastating disease that causes the inflammation of the meninges and the subarachnoid space and accounts for high rates of morbidity and mortality worldwide [1]. Although the incidence of bacterial meningitis in Western countries has gradually declined from 3 to 4% to 0.7 to 0.9 per 100,000 persons per year over the past 10 to 20 years, in African countries, the incidence rates are steadily rising at 10 to 40 cases per 100,000 persons per year [2]. In addition to acute complications, approximately 10 to 27% of meningitis survivors also suffer from lingering neurological consequences, such as cognitive impairment, including dementia [3]. However, the mechanisms that cause long-term cognitive impairment after meningitis remain elusive.

Evidence suggests a strong association between inflammation and cognitive decline in several models [4]. Microglia, the primary innate immune cells in the central nervous system (CNS), play a critical role in regulating the brain’s inflammatory milieu during healthy and diseased conditions [5]. During CNS insult, the activated microglia transform into an amoeboid phenotype and respond to the pathologic event by releasing a plethora of substances, including cytokines, chemokines, and growth factors [6]. Understanding the activation of microglia is essential, as studies suggest that the activation of microglia could perpetrate neurodegeneration through several mechanisms [7]. For the past few decades, visualizing microglial activation in vivo, after CNS insult, has been enabled by positron emission tomography (PET), an ideal imaging technique. The noninvasive detection of neuroinflammation by PET imaging is used in a broad spectrum of diseases, including neurodegenerative disorders [8, 9] and psychiatric complications [10, 11], by utilizing the pharmacological ligands of the mitochondrial translocator protein (TSPO). In eukaryotes, TSPO, previously known as the peripheral benzodiazepine receptor (PBR), is ubiquitously expressed in the outer mitochondrial membrane in astrocytes and microglial cells. The expression of the neuroimmunomodulatory target TSPO rises after CNS insult due to the presence of infiltrating inflammatory cells and activated microglia [12, 13]. Interacting with voltage-dependent anion channel (VDAC) and the adenine nucleotide translocator (ANT), TSPO modulates the mitochondrial permeability transition pore (MPTP), leading to either cell death via the opening of MPTP or cell protection by blocking MPTP and thereby regulating mitochondrial function [14, 15].

In this context, we hypothesize that persistent microglial activation and perpetuated levels of TSPO expression might play a role in cognitive decline in meningitis survivors. Previously, we reported long-term cognitive impairment after meningitis induced by *Escherichia coli* [16], *Klebsiella pneumoniae* [17], *Streptococcus agalactiae* [18], and *Streptococcus pneumoniae* [19] in neonatal and adult rats. However, in vivo microglial activation and TSPO levels were not explored to evaluate the mechanistic and diagnostic significance of PET imaging. Hence, we aimed to investigate in vivo microglial activation 24 h (acute) and 10 days (long-term) after experimental meningitis induction as measured by [^11^C]PBR28 PET/CT imaging. Additionally, we sought to compare the levels of oxidative stress markers, inflammatory mediators, and markers of glial activation in the 24-h and the 10-day groups. We used a battery of behavioral tests to assess habituation memory, aversive memory, and long-term memory, 10 days after meningitis induction. Due to the lack of evidence on the TSPO-mediated mitochondrial pathway after meningitis, we also investigated the cardiolipin, cytochrome-*c*, and caspase levels in the experimental meningitis models.

## Materials and methods

### Infectious organism used for the induction of meningitis

The strain used to induce meningitis, namely serotype III *S. pneumoniae*, was cultured in 5 mL of Todd Hewitt Broth BBL™, diluted in fresh medium, and then grown to the logarithmic phase. The culture was centrifuged for 10 min at 1200 rpm and resuspended in sterile pyrogen-free saline to a concentration of 5 × 10^9^ colony-forming units (CFU) [20].

### Animal model of meningitis

Eight-week-old male Wistar rats, weighing 200 to 250 g, purchased from Charles River, were housed at a temperature of 20 °C and a humidity level of 30% on a 12-h light-dark cycle (lights on 0600 hours). Food and water were available ad libitum. All protocols were approved by the Institutional Animal Welfare Committee (AWC) of the Center for Laboratory Animal Medicine and Care (CLAMC) of the University of Texas Health Science Center at Houston (UTHealth), TX, USA (AWC-15-0099). All possible efforts were made to reduce animal suffering and the number of animals used. All bacterial inoculations were performed under anesthesia using 3% isoflurane. The animals received an intracisternal (i.c.) injection of 10 μL of artificial cerebrospinal fluid (CSF) as a placebo or an equivalent volume of *S. pneumoniae* suspension. The animals received fluid replacement soon after bacterial inoculation and were then returned to their cages. Meningitis was confirmed by incubating a quantitative culture of 5 μL of CSF at 35 °C with 5% CO_2_ in sheep blood agar [19]. After 18 h of meningitis induction or artificial CSF injection, all animals received ceftriaxone (100 mg/kg, i.p.) twice a day for 7 days [21].

### Study design

After 1 week of acclimatization, the rats were grouped into the 24-h or 10-day group. The groups were 24 h control, 24 h meningitis, 10 days control, and 10 days meningitis. Twenty-four hours and 10 days after meningitis induction, all rats were subjected to PET imaging. Rats from the 10-day groups were subjected to behavioral tasks before they underwent PET/CT. We used a separate cohort of rats for each of the different behavioral studies performed. Following PET imaging, rats were euthanized (under 4 to 5% isoflurane) and transcardially perfused with saline followed by 4% paraformaldehyde to collect the brains for immunohistochemistry. For biochemical evaluation, the prefrontal cortex (PFC) and hippocampus were immediately isolated and stored at − 80 °C until further processing.

### Small animal PET/CT scan

Control rats and meningitis rats from both the 24-h and 10-day groups were subjected to PET/CT imaging. All rats were manipulated in accordance with the University of Texas MD Anderson department’s IACUC guidelines. To perform the imaging, rats were anesthetized with 2% isoflurane and PET imaging was performed in a dedicated small animal Bruker Albira PET/SPECT/CT scanner (Bruker Biospin Corp., Billerica, MA, USA) with a 10-cm and a 12-cm axial field-of-view (FOV) and transaxial FOV, respectively. An intravenous bolus injection of ~ 300 μCi of [^11^C]PBR28 in 200 μL saline was given concomitantly via the tail vein along with the acquisition of PET imaging in the list-mode. The PET scan was acquired for 20 min, followed by a CT scan (400 μA, 45 kV, 120 projections) of the head and upper torso for attenuation correction and image registration. The list-mode data were binned into dynamic frames: 15 × 20 s, 5 × 60 s, and 2 × 300 s. The PET images were then reconstructed using the maximum likelihood expectation maximization (MLEM) method with 12 iterations. Scatter, random, decay, and attenuation corrections were applied. The analysis was performed with Pmod (PMOD Technologies Ltd., Zürich, Switzerland). The mean standard uptake volume (SUV)-body weight (g/ml) of the whole brain and normal muscle tissue were measured [22].

### Biochemical evaluation

#### Oxidative stress parameters

##### Nitrite/nitrate concentration

The tissue samples were homogenized in 1 ml of ice-cold 0.1 M phosphate buffer (pH 7.4). In addition, an aliquot of homogenate was centrifuged at 10000 rpm for 8 min with trichloroacetic acid (TCA) 50% to deproteinization of the sample. Nitrite/nitrate concentration was assayed spectrophotometrically using Griess reagents [1% sulfanilamide in 5% phosphoric acid and 0.1% *N*-1-naphthylethylenediamine dihydrochloride in distilled H_2_O (NED solution)] and vanadium (III) chloride. A standard curve was run simultaneously with each set of samples, and the optical density was measured spectrophotometrically at 550 nm [23]. The results were expressed as nmol/mg of protein.

##### Myeloperoxidase (MPO) activity

The tissue samples were homogenized in 1 ml of ice-cold 0.1 M phosphate buffer (pH 7.4) and 0.5% hexadecyltrimethylammonium bromide (Sigma-Aldrich, Saint Louis, USA). Homogenates were centrifuged at 15000 rpm for 15 min at 4 °C and were used an aliquot of supernatant that was mixed with a solution of 1.6 mM tetramethylbenzidine (TMB) and 1 mM H_2_O_2_. The supernatant was mixed with a solution of 1.6 mM tetramethylbenzidine (TMB) and 1 mM H_2_O_2_. MPO activity was measured spectrophotometrically at 650 nm at 37 °C [24]. The results were expressed as mU/mg of protein.

##### Lipid peroxidation

As an index of oxidative damage to lipids, we verified the formation of thiobarbituric acid reactive species (TBARS) in an acid-heating reaction. The tissue samples were homogenized in 1 ml of ice-cold 0.1 M phosphate buffer (pH 7.4). Then, an aliquot of homogenate was centrifuged at 1000 rpm for 10 min with trichloroacetic acid (TCA) 10% to deproteinization. The pellet was discarded and aliquots of supernatants were separated and used for the determination of lipid oxidative damage. TBARS was determined by the absorbance at 535 nm using 1,1,3,3-tetramethoxypropane as an external standard [25]. The results were expressed as malondialdehyde (MDA) equivalents nmol/mg of protein.

##### Protein carbonyl formation

The tissue samples were homogenized in 1 ml of ice-cold 0.1 M phosphate buffer (pH 7.4). Homogenates were centrifuged at 14000 rpm for 15 min at 4 °C, and supernatants were used for the determination of protein oxidative damage. Protein oxidation was assessed by the determination of carbonyl groups based on the reaction with dinitrophenylhydrazone. Briefly, proteins were precipitated by the addition of 20% trichloroacetic acid and were dissolved in dinitrophenylhydrazone, and the absorbance was read at 370 nm [26]. The results were expressed as protein carbonylation nmol/mg of protein.

##### Superoxide dismutase (SOD) activity

The tissue samples were homogenized in 1 ml of glycine buffer (pH 10.2). Homogenates were centrifuged at 3000 rpm for 10 min at 4 °C, and supernatants were used for the determination of SOD activity. SOD (EC 1.15.1.1) activity was determined using a spectrophotometric assay based on the superoxide-dependent oxidation of epinephrine to adrenochrome at 32 °C. Absorption was measured at 480 nm [27]. The SOD-specific activity was represented as mU/mg of protein.

##### Catalase (CAT) activity

The tissue samples were homogenized in 1 ml of ice-cold 0.1 M phosphate buffer (pH 7.4). Homogenates were centrifuged at 3.000 rpm for 10 min at 4 °C. The pellet was discarded, and aliquots of supernatants were separated and used for the determination of catalase. CAT (EC 1.11.1.6) activity was assayed by measuring the decrease in absorbance at 240 nm in a reaction medium containing 20 mM H_2_O_2_, 0.1% Triton X-100, 10 mM potassium phosphate buffer, at a pH 7.0, and supernatants containing 0.1–0.3 mg protein/mL [28]. The specific activity was expressed as mU/mg of protein.

### Immunoblot analysis

#### Protein determination

The results were normalized by the protein content, following Lowry’s method using bovine serum albumin (BSA) as a standard. The absorbance was read at 700 nm, and the results were expressed as mg of protein [29].

#### Isolation of mitochondria

Mitochondrial isolation was performed according to the manufacturer’s instructions (Catalog no. 89801, Thermo Scientific^TM^, USA). Briefly, the tissue (50–200 mg) was disrupted using a homogenizer in PBS and then centrifuged at 1000×*g* for 3 min at 4 °C. The pellet was suspended in 800 μL BSA/reagent A solution, then the suspension was vortexed at medium speed for 5 s and incubated on ice for exactly 2 min afterward. Then, 10 μL of mitochondria isolation reagent B was added, and it was vortexed at maximum speed for 5 s. Approximately 800 μL of mitochondria isolation reagent C was added to the tube, which was then inverted several times and centrifuged at 700×*g* for 10 min at 4 °C. The supernatant was then centrifuged at 3000×*g* for 15 min at 4 °C. The mitochondrial pellet was collected and washed.

#### Western blot analysis

Western blot analysis was performed as per the protocols of previous studies [30]. The PFC and hippocampus regions of the rat brains were thawed and homogenized using Complete Protease Inhibitor Cocktail tablets (Roche, Diagnostics, IN, USA). The homogenate was centrifuged at 12000 rpm for 20 min at 4 °C. Protein concentrations in tissue were determined using the bicinchoninic acid (BCA) assay method. For the western blot run, equal amounts of proteins (30-50 μg) for each sample were loaded in Mini-Protean TGX precast gels (Bio-Rad, CA, USA). Proteins were transferred onto polyvinylidenedifluoride (PVDF) membranes using a Trans-Blot® Turbo™ system (Bio-Rad, CA, USA). Then, the PVDF membranes were blocked with 5% nonfat dry milk (Bio-Rad) in Tris-buffered saline plus 0.1% Tween 20 buffer (TBST, Bio-Rad, CA, USA) for 1 h at room temperature (RT) and kept overnight in a cold room on a shaker with primary antibodies afterward. The primary antibodies used for mitochondrial fractions were TSPO (1:500, LS Bio, LS-B14234), VDAC (1:1000, Thermo, PA1-954A), and ANT (1:1000, Abcam, ab102032). The primary antibodies used in tissue homogenate were glial fibrillary acidic protein (GFAP) (1:1000, Abcam, ab7260), CD 11B (1:1000, Abcam, ab75476), ionized calcium-binding adaptor molecule (IBA)-1 (1:1000, Abcam, ab108539), anti-oligodendrocyte (Oligo, 1:1000, Abcam, ab53041), NeuN (1:1000, Abcam, ab177487), and cytochrome-*c* (1:1000, Abcam, ab13575). The following day, the blots were washed three times in TBST and incubated with a horseradish peroxidase-conjugated secondary antibody (1:10000) for 1 h at RT. After three final washes of 10 min each with TBST, bands were detected using enhanced chemiluminescence (Clarity Western ECL Substrate; Bio-Rad, CA, USA) with the ChemiDoc MP (Bio-Rad, CA, USA) western blotting imaging system. After imaging, the blots were incubated in the stripping buffer (Thermo Fisher Scientific; 46430, IL, USA) for 10–15 min at RT followed by three washes with TBST. The stripped blots were incubated in blocking solution (5% nonfat dry milk in TBST) for 1 h and then incubated with the primary antibody directed against β-tubulin (1:5000, Abcam, ab6046) or COX IV (1:5000, Abcam, ab16056) as a loading control. Densitometric analysis of each protein was conducted using Image Lab™ software (Bio-Rad, CA, USA). The results were expressed as the ratio between the loading control and the target protein.

### Multiplex assay for the quantification of the inflammatory cytokines

Cytokine levels were assayed using multiplex fluorescent immunoassay kits (Bio-Plex Pro™ Rat Cytokine 14-Plex Assay) [31]*.* The xMAP platform used here was based on rules-based medicine (RBM) fluorescent beads and antibody pairs. These are sensitive, specific, and widely used reagents, produced by manufacturers, and data collected using xMAP multiplex beads have been widely reported in the literature, in studies in which multiple proteins are assayed simultaneously. Tissue lysates were prepared according to the instructions provided by the Bio-Plex Cell lysis kit (#171304011) with a protease inhibitor cocktail (Sigma-Aldrich, St. Louis, MO, USA), followed by centrifugation at 4 °C for 10 min at 10000×*g*. The assays were conducted in 96-well polystyrene, round-bottom microplates. Initially, a 50 μL aliquot of the working bead mixture was transferred into the wells, and the plate was washed two times by adding 100 μL of assay buffer into each well. Then, 50 μL of the standard, control, or total extracts were added to each well. The plate was incubated on a plate shaker (850 rpm) in the dark at RT for 60 min. The plate was then placed in the magnetic separator and incubated for separation for 60 s. The supernatant was carefully removed from each well by manual inversion. Beads were then washed three times by adding 100 μL of assay buffer into each well, to ensure the removal of any undesirable or nonspecifically bound antibodies. After this protocol, 25 μL of a detection antibody was added to each well. Incubation was again conducted in the dark and at RT on a plate shaker (850 rpm) for 30 min, and washing was performed as previously described. Finally, 50 μL of streptavidin-PE was added to each well. The plate was incubated on a plate shaker (850 rpm) in the dark at RT for 10 min. After the magnetic separation of the beads, the supernatant was carefully removed by manual inversion, and washing was performed as previously described. Assay buffer (125 μL) was added to each well, and the plate was placed onto a plate shaker for approximately 30 s to achieve gentle agitation of the beads. Samples were run in duplicates using a Bio-Plex system (Bio-Plex 200 Systems, Bio-Rad, Hercules, CA), and data analysis was conducted in Bio-Plex Manager 4.0 using a five-parameter logistic regression model.

### Immunofluorescence

The rats were anesthetized with isoflurane and were transcardially perfused with PBS followed by 4% paraformaldehyde to fix the brain. For the immunofluorescence (IF) assay, the brain was fixed in 4% buffered formaldehyde solution and embedded in optimum cutting temperature (OCT). The coronal sections were cut to 8 μm and blocked in 3% BSA and horse serum. After blocking, the sections were incubated overnight with antibodies for IBA-1, unconjugated, rabbit polyclonal with reactivity to human, mouse, cat, and rat (1:1000, Wako, 019-19741) and GFAP, unconjugated, rabbit polyclonal with reactivity to human, mouse, cat, and dog (1:1000, Abcam, ab7260). The next day, after washing three times with PBS, goat anti-rabbit secondary antibody, Alexa Fluor Plus 488 (1:1000, Invitrogen, A13201) and to stain nuclei DAPI were applied for each section. To ensure the labeling in the experiment, we performed the primary antibody controls, secondary antibody controls, and label (endogenous tissue background) controls. The tissue sections were imaged using the confocal laser scanning microscopy platform Leica TCS SP8. We used the ImageJ software to count the number of cells from confocal images (https://imagej.nih.gov/ij/).

### ELISA

The assays for caspase-3 (MBS018987), caspase-9 (MBS088765), and cardiolipin (MBS721201) were performed using an ELISA kit as recommended by the manufacturers.

### Behavioral assessment

#### Open-field task

The apparatus was a 40 × 60 cm open field surrounded by 50-cm-high dark gray walls with a front glass wall. Black lines divided the floor of the open field into nine rectangles. Each rat was gently placed in the center of the open field and was allowed to explore the arena for 5 min (training session; on day 9). The number of crossings (i.e., the number of times that the animal crossed the black lines) and rearing movements (exploratory behavior) were measured. Immediately after this task, the animals were returned to their home cages. During the test session, which was performed 24 h after the training session, all rats were subjected to a second open-field task (on day 10). In both sessions, crossings and rearing behaviors were counted for 5 min. A reduction in the number of crossings and rearings across the two sessions was taken as a measure of memory retention [32].

#### Step-down inhibitory avoidance task

The dimensions of the acrylic box used to test inhibitory avoidance were 50 × 25 × 25 cm (Albarsch, Porto Alegre, Brazil). The floor was made up of parallel stainless steel bars (1-mm diameter) spaced 1 cm apart. A 7-cm-wide, 2.5-cm-high platform was placed on the floor of the box against the left wall. During the training trial (on day 9), animals were placed on the platform, and their latency to step-down on the grid with all four paws was measured. Immediately after stepping down on the grid, the animals received a 0.4-mA, 2.0-s footshock and were then returned to their home cage. Retention or test trial was performed 24 h after training (on day 10). The retention trial was identical to the training trial, except that no footshock was given. The latency time to step-down (maximum, 180 s) was used as a measure of inhibitory avoidance retention [33].

#### Novel object recognition test

On day 8, after the induction of meningitis, rats were subjected to a novel object recognition (NOR) test. The first day was dedicated to a habituation session. Animals were placed in a NOR chamber in an open field (60 × 40 cm) surrounded by 50-cm-high plexiglass walls and were allowed to explore freely for 5 min. On day 9, rats were exposed to two identical objects for 10 min (acquisition trial). The objects used in this experiment were square wooden cases of the same color. The heights of the objects were comparable, and the objects were heavy enough to ensure that the animals would not displace them. On the following day (day 10), one of the square wooden cases was replaced by a novel object, and the rat was allowed to explore the familiar and novel object in the test box for 5 min (retention trial) [34]. The recognition index was calculated for each animal and was reported as the ratio TB/(TA + TB) (TA = time spent exploring the familiar object, A; TB = time spent exploring the novel object, B).

### Statistical analysis

Data from the open-field task groups were compared using paired Student’s *t* test and analysis of variance followed by Tukey’s post hoc test. Comparisons among groups in the step-down inhibitory avoidance task were performed using a Mann–Whitney *U* test. Intragroup comparisons were performed using the Wilcoxon signed-rank test. For all the analyses, the results were expressed as the mean ± SEM. The differences between groups were analyzed by unpaired Student’s *t* tests. The statistically significant results are indicated by *P < 0.05. All statistical analyses were performed using GraphPad Prism 7.0 (GraphPad Software, Inc., La Jolla, CA, USA).

## Results

### Increased in vivo microglial activation evidenced by elevated [^11^C]PBR28 uptake

**Fig. 1:**
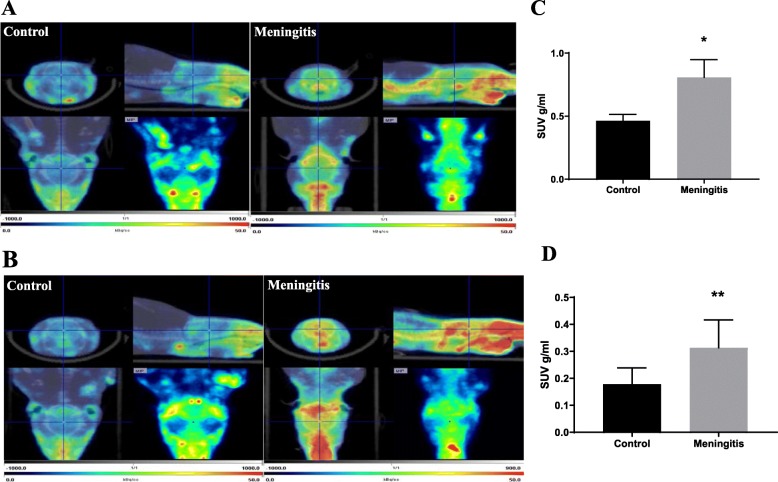
In vivo PET imaging of TSPO using [^11^C]PBR28. Representative summed images from the dynamic reconstruction of simultaneously acquired PET/CT scans from 10-week-old male Wistar rats subjected to experimental meningitis and controls, 60 min after intravenous injection of [^11^C]PBR28. The coronal, sagittal, and dorsal orientations of the brain are presented, showing increased brain [^11^C]PBR28 uptake in the experimental meningitis model than in controls. **a** Twenty-four hours after experimental meningitis induction. **b** Ten days after experimental meningitis induction. The SUV scale represents the standardized uptake value (SUV). Quantification of [^11^C]PBR28 uptake detected in the experimental meningitis model was significantly higher than that in the controls based on measures of brain/muscle. **c** SUV 24 h after experimental meningitis induction. **d** SUV 10 days after experimental meningitis induction. The results are expressed as the mean ± SEM for *n* = 5–8 rats. *P < 0.05, **P < 0.01 compared to controls

To investigate whether microglia are activated during acute meningitis, animals were subjected to [^11^C]PBR28, a TSPO-PET tracer that has been widely used for both preclinical and clinical investigations [35, 36]. We subjected both 24-h and 10-day groups to a TSPO-PET scan followed by CT imaging. The SUV was calculated using the following formula: SUV = *r*/(*a*'/*w*), where *r* is the radioactivity concentration expressed as kBq/ml, which is measured by the PET scanner within a region of interest (ROI); *a*' is the injected radiolabeled tracer (decay-corrected) expressed as kBq; and *w* is the weight of the animal. As shown in Fig. 1, the whole brain uptake of [^11^C]PBR28 was significantly increased in rats, 24 h (*P* < 0.05) and 10 days (*P* < 0.01) after the induction of meningitis. The increase in [^11^C]PBR28 uptake suggests the overexpression of TSPO and increased microglial activation after experimental meningitis. Persistent microglial activation was present, as demonstrated by the increased uptake of the radiotracer even after 10 days when the animals were free from infection.

### Elevated levels of oxidative stress markers observed after experimental meningitis

**Fig. 2:**
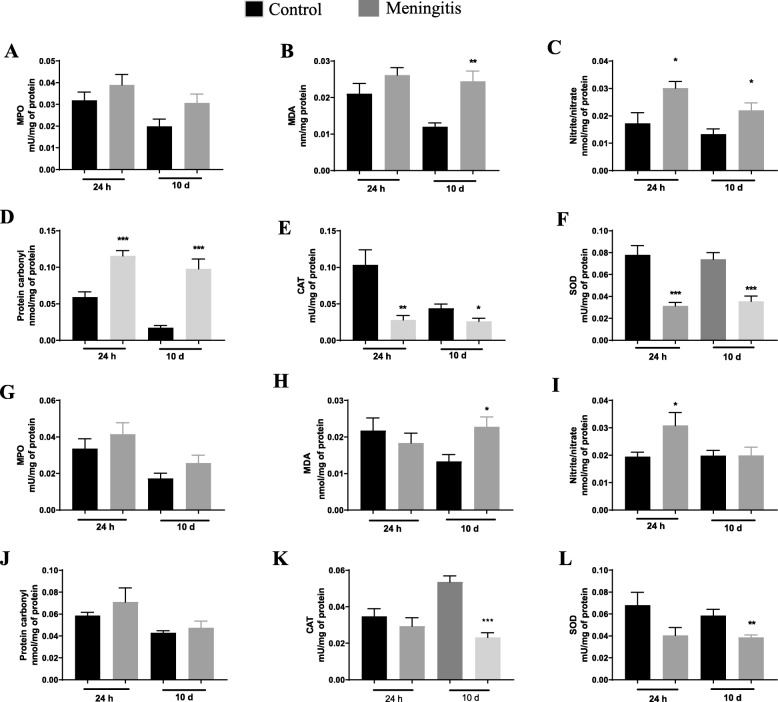
Elevated oxidative stress and reduced enzymatic defense after experimental meningitis. PFC levels of **a** MPO, **b** TBARS, **c** nitrite/nitrate, **d** protein carbonyl, **e** CAT, and **f** SOD in experimental meningitis and control animals 24 h and 10 days after meningitis induction. Hippocampal levels of **g** MPO, **h** TBARS, **i** nitrite/nitrate, **j** protein carbonyl, **k** CAT, and **l** SOD in experimental meningitis and control animals 24 h and 10 days after meningitis induction. The results are expressed as the mean ± SEM for *n* = 6 rats. *P < 0.05, **P < 0.01, **P < 0.001 compared to controls

We have previously demonstrated increased levels of oxidative stress parameters in experimental meningitis models induced by *E. coli K1*, *S. agalactiae*, and *S. pneumoniae* [16, 18, 37]. In this study, oxidative damage in the PFC and hippocampus were evaluated in both the 24-h and 10-day groups (Fig. 2). We found no significant changes in MPO activity in both PFC and hippocampus in either the 24-h or10-day groups. However, we observed a significant increase in lipid peroxidation marker, TBARS, levels in the PFC (*P* < 0.01), and hippocampus (*P* < 0.05) in the meningitis group compared to the control group. In the PFC, elevated nitrite/nitrate concentrations (*P* < 0.05 and *P* < 0.05, respectively) and protein carbonyl levels (*P* < 0.001 and *P* < 0.001, respectively) were observed in both 24-h and 10-day groups. In the hippocampus, only the 24-h group demonstrated elevated nitrite/nitrate concentrations (*P* < 0.05), but no significant change was observed in the 10-day group for either nitrite/nitrate concentrations or protein carbonyl levels. As expected, the experimental meningitis group showed decreased CAT levels in the PFC in both the 24-h (*P* < 0.01) and 10-day (*P* < 0.05) groups. Only the 10-day group showed a significant decrease in CAT levels (*P* < 0.001) in the hippocampus. Levels of another antioxidant enzyme, SOD, were also decreased in the PFC in both the 24-h (*P* < 0.001) and 10-day (*P* < 0.001) groups. We also found decreased levels of SOD in the hippocampus of the 10-day (P < 0.01) group animals (Fig. 2). The meningitis group demonstrated a decrease in complex III activity in the hippocampus only in the 24-h group. There was no significant change in complex I, II, and IV activity in either the 24-h or the 10-day group (data not shown).

### Increased levels of inflammatory mediators after experimental meningitis

The first line of defense in response to *S. pneumoniae* bacterial invasion during meningitis is achieved by the host immune response and the production of inflammatory mediators [38]. Previously, we have reported a kinetic study of cytokine and chemokine release after experimental meningitis [18]. In the current study, 24 h and 10 days after meningitis, the expression of cytokines and chemokines (IL1-α, IL1-β, IL-4, IL-6, IL-7, IL-10, IL-12, IL-13, IL-17, IL-18, TNF-α, and INF-γ) was evaluated using the Multiplex assay (Tables 1 and 2). As expected, in the PFC, we observed increased levels of pro-inflammatory cytokines such as IL1-α, IL1-β, IL-6, IL-17, TNF-α, and INF-γ (*P* < 0.05) and decreased levels of IL-7, IL-10, and IL-13 (*P* < 0.05) in the 24-h meningitis group (Table 1). In the hippocampus, we observed increased levels of pro-inflammatory cytokines, such as IL1-α, IL1-β, IL-6, IL-18, TNF-α, and INF-γ (*P* < 0.05) in the 24-h meningitis group (Table 1). Although the CSF tested negative for organisms (data not shown) 10 days after the infection, the levels of TNF-α (*P* < 0.05) were still high in the PFC compared to the respective control groups, with a simultaneous decrease in IL-12 and IL-17 (*P* < 0.05) (Table 2). We also observed increased IL-4 levels (*P* < 0.05) and decreased IL-17 levels (*P* < 0.05) in the meningitis rat hippocampus, 10 days after the infection (Table 2).

**Table 1:** Increased inflammatory mediator levels 24 h after experimental meningitis induction in the PFC and hippocampus. The results are expressed as the mean ± SEM for *n* = 5–6 rats

Cytokines (pg/μg)	PFC—24 h		Hippocampus—24 h	
	Control	Meningitis	Control	Meningitis
IL-1a	0.018 ± 0.001	0.711 ± 0.222*	0.014 ± 0.001	0.199 ± 0.020*
IL-1β	0.165 ± 0.009	1.569 ± 0.256*	0.107 ± 0.008	0.267 ± 0.027*
IL-4	0.009 ± 0.001	0.007 ± 0.001	0.005 ± 0.0004	0.005 ± 0.0009
IL-5	0.052 ± 0.003	0.046 ± 0.009	0.034 ± 0.002	0.315 ± 0.003
IL-6	0.077 ± 0.005	0.984 ± 0.030*	0.059 ± 0.005	0.447 ± 0.050*
IL-7	0.010 ± 0.0007	0.007 ± 0.0008*	0.003 ± 0.0002	0.002 ± 0.0008
IL-10	0.033 ± 0.004	0.019 ± 0.001*	0.022 ± 0.001	0.023 ± 0.001
IL-12	0.013 ± 0.001	0.006 ± 0.008	0.009 ± 0.001	0.009 ± 0.0008
IL-13	0.027 ± 0.001	0.012 ± 0.0008*	0.012 ± 0.0008	0.011 ± 0.001
IL-17	0.021 ± 0.002	0.041 ± 0.001*	0.011 ± 0.0002	0.014 ± 0.001
IL-18	0.037 ± 0.003	0.050 ± 0.006	0.031 ± 0.002	0.043 ± 0.003*
TNF-α	0.054 ± 0.004	0.312 ± 0.029*	0.041 ± 0.005	0.059 ± 0.003*
INF-γ	0.021 ± 0.002	0.070 ± 0.006*	0.023 ± 0.002	0.069 ± 0.006*

*P < 0.05 compared to controls

**Table 2:** Increased inflammatory mediator levels 10 days after experimental meningitis induction in the PFC and hippocampus. The results are expressed as the mean ± SEM for *n* = 5–6 rats

Cytokines (pg/μg)	PFC—10 days		Hippocampus—10 days	
	Control	Meningitis	Control	Meningitis
IL-1a	0.1083 ± 0.012	0.133 ± 0.016	0.082 ± 0.013	0.096 ± 0.014
IL-1β	0.347 ± 0.029	0.316 ± 0.026	0.212 ± 0.015	0.175 ± 0.009
IL-4	0.009 ± 0.001	0.006 ± 0.001	0.010 ± 0.001	0.022 ± 0.002*
IL-6	0.423 ± 0.048	0.480 ± 0.067	0.198 ± 0.031	0.199 ± 0.025
IL-10	0.084 ± 0.006	0.110 ± 0.016	0.029 ± 0.004	0.031 ± 0.004
IL-12	0.192 ± 0.022	0.085 ± 0.011*	0.08 ± 0.013	0.086 ± 0.009
IL-17	0.088 ± 0.008	0.043 ± 0.008*	0.040 ± 0.006	0.019 ± 0.003*
IL-18	0.043 ± 0.004	0.039 ± 0.004	0.077 ± 0.011	0.073 ± 0.004
TNF-α	0.022 ± 0.001	0.032 ± 0.002*	0.019 ± 0.002	0.019 ± 0.001
INF-γ	0.093 ± 0.007	0.101 ± 0.006	0.082 ± 0.008	0.074 ± 0.004

*P < 0.05 compared to controls

### Upregulation of glial cell activation after experimental meningitis

**Fig. 3:**
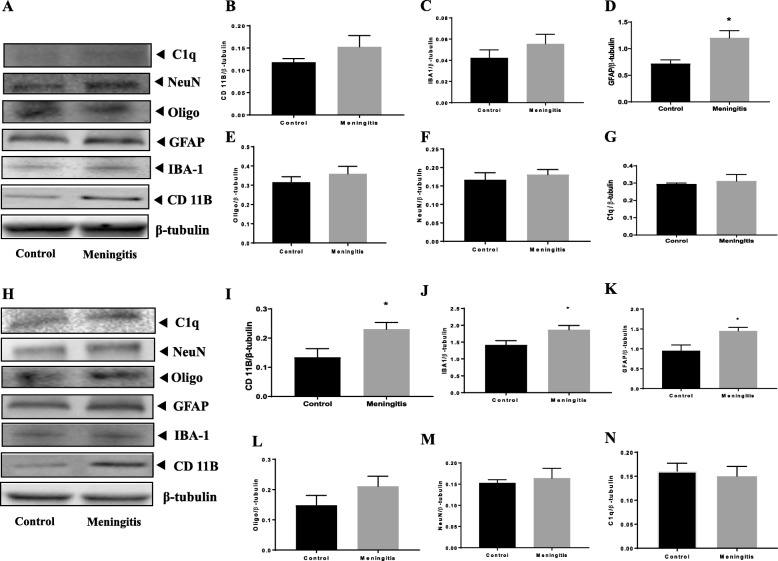
Upregulation of glial markers 24 h after experimental meningitis induction in the PFC and hippocampus. Western blot analysis of the tissue protein levels of glial and neuronal markers. Quantification of immunoblot data using the densitometric analysis of each protein was carried out using Image Lab™ software (Bio-Rad, CA, USA). Representative immunoblots and quantification of CD 11B, IBA-1, GFAP, Oligo, NeuN, C1q, and β-tubulin in the PFC (**a**–**g**) and hippocampus (**h**–**n**). The values for all protein levels were normalized to those of β-tubulin. The results are expressed as the mean ± SEM for *n* = 4–5 rats. *P < 0.05 compared to controls

**Fig. 4:**
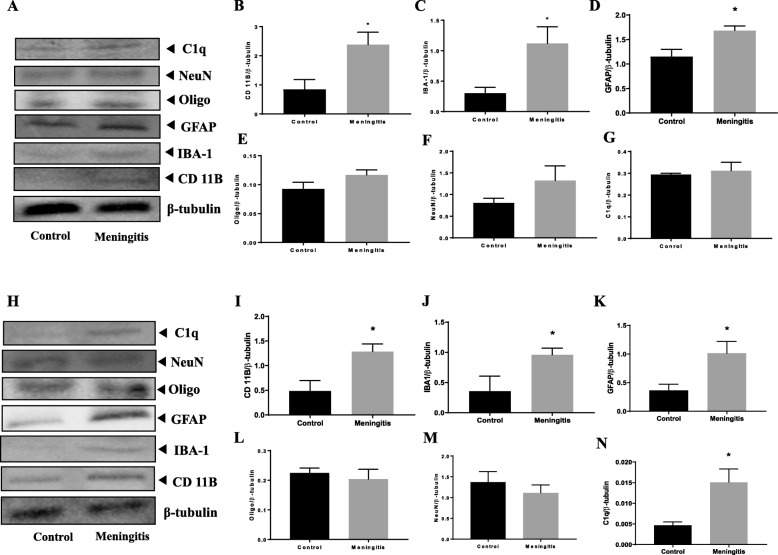
Upregulation of glial markers 10 days after experimental meningitis induction in the PFC and hippocampus. Western blot analysis of the tissue protein levels of glial and neuronal markers. Quantification of the immunoblot data using the densitometric analysis of each protein was carried out using Image Lab™ software (Bio-Rad, CA, USA). Representative immunoblots and quantification of CD 11B, IBA-1, GFAP, Oligo, NeuN, C1q, and β-tubulin in the PFC (**a**–**g**) and hippocampus (**h**–**n**). The values for all protein levels were normalized to those of β-tubulin. The results are expressed as the mean ± SEM for *n* = 4–5 rats. *P < 0.05 compared to controls

**Fig. 5:**
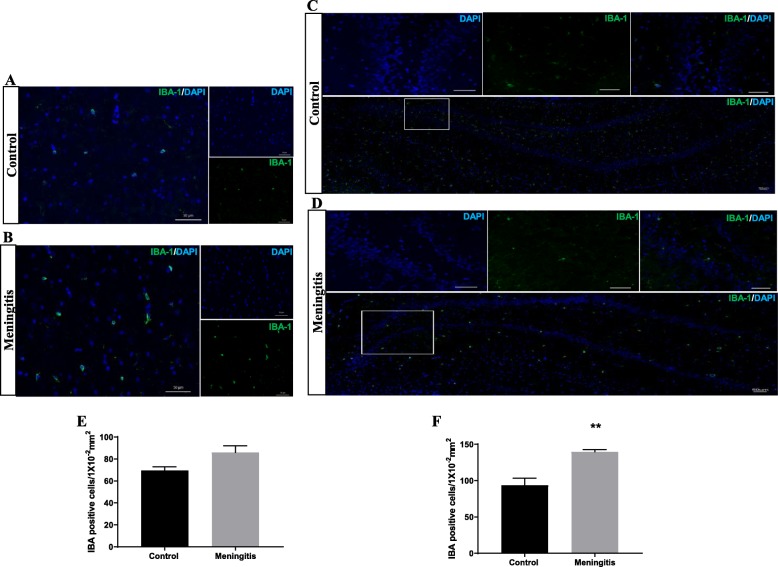
Increased microglial activation 24 h after experimental meningitis induction. Immunofluorescence analysis for IBA-1-positive cells in experimental meningitis rats and control rats. **a**, **b** Representative microscopic field images (magnification, × 400) immunostained with IBA-1 antibodies in the PFC for 24 h. **c**, **d** Representative microscopic field images (magnification, × 200) immunostained with IBA-1 antibodies in the hippocampus for 24 h. The number of IBA-1-positive cells/1 × 10^−2^ mm^2^ in the **e** PFC and **f** hippocampus after 24 h. The results are expressed as the mean ± SEM for *n* = 4–5 rats. **P < 0.01 compared to controls

**Fig. 6:**
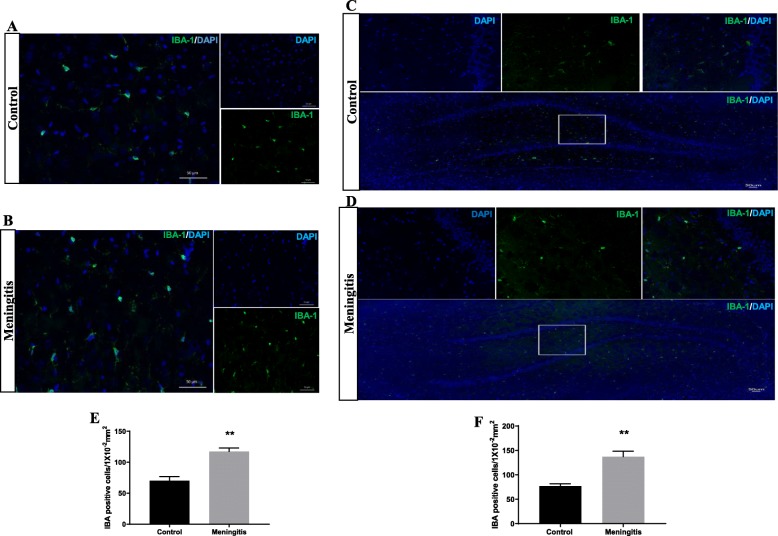
Increased microglial activation 10 days after experimental meningitis induction. Immunofluorescence analysis for IBA-1-positive cells in experimental meningitis rats and control rats. **a**, **b** Representative microscopic field images (magnification, × 400) immunostained with IBA-1 antibodies in the PFC for 24 h. **c**, **d** Representative microscopic field images (magnification, × 200) immunostained with IBA-1 antibodies in the hippocampus for 24 h. The number of IBA-1-positive cells/1 × 10^−2^ mm^2^ in the **e** PFC and **f** hippocampus after 24 h. The results are expressed as the mean ± SEM for *n* = 4–5 rats. **P < 0.01 compared to controls

**Fig. 7:**
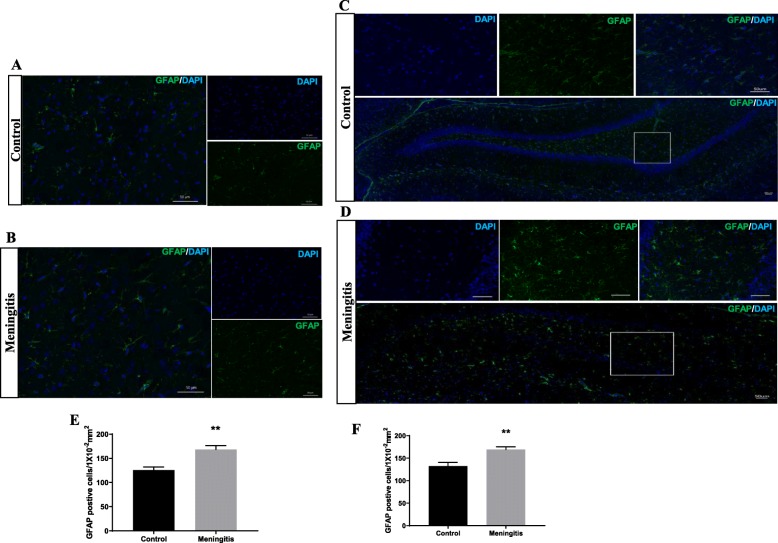
Increased astroglial activation 24 h after experimental meningitis induction. Immunofluorescence analysis for GFAP-positive cells in experimental meningitis rats and control rats. **a**, **b** Representative microscopic field images (magnification, × 400) immunostained with GFAP antibodies in the PFC for 24 h. **c**, **d** Representative microscopic field images (magnification, × 200) immunostained with GFAP antibodies in the hippocampus for 24 h. The number of GFAP-positive cells/1 × 10^−2^ mm^2^ in the **e** PFC and **f** hippocampus after 24 h. The results are expressed as the mean ± SEM for *n* = 4–5 rats. **P < 0.01 compared to controls

**Fig. 8:**
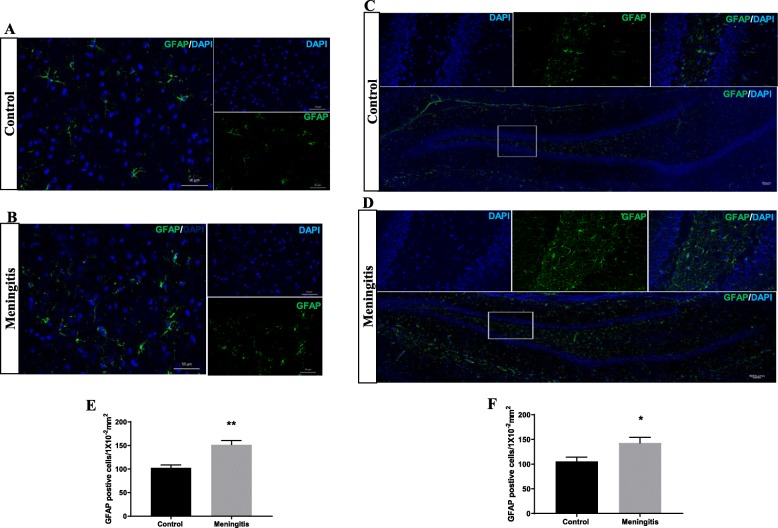
Increased astroglial activation 10 days after experimental meningitis induction. Immunofluorescence analysis for GFAP-positive cells in experimental meningitis rats and control rats. **a**, **b** Representative microscopic field images (magnification, × 400) from the 10-day group immunostained with GFAP antibodies in the PFC. **c**, **d** Representative microscopic field images (magnification, × 200) from the 10-day group immunostained with GFAP in the hippocampus. The number of GFAP-positive cells/1 × 10^−2^ mm^2^ in **e** PFC and **f** hippocampus in the 10-day group. The results are expressed as the mean ± SEM for *n* = 4–5 rats. *P < 0.05, **P < 0.01 compared to controls

To understand the glial contribution after meningitis, we evaluated the levels of microglial (IBA-1, CD 11B), astrocyte (GFAP), oligodendrocyte (Oligo), and neuronal (NeuN) markers 24 h and 10 days after experimental meningitis induction. In the meningitis group, we observed increased expression of GFAP (*P* < 0.05), a marker of astrocytes, 24 h after infection, with no significant change in IBA-1, Oligo, and NeuN levels in the PFC (Fig. 3). However, in the hippocampus, we found a significant increase in the expression of microglial markers IBA-1 (*P* < 0.05) and CD 11B (*P* < 0.05) and astrocyte marker GFAP (*P* < 0.05) in the meningitis group after 24 h of infection (Fig. 3). We observed increased expression of IBA-1 (*P* < 0.05), CD 11B (*P* < 0.05), and GFAP (*P* < 0.05) 10 days after infection, with no significant changes in Oligo or NeuN levels in either the PFC or the hippocampus (Fig. 4). We also observed increased expression of C1q, the first subcomponent of the C1 complex in the classical pathway of complement activation, 10 days after meningitis induction (Fig. 4), but no change was observed 24 h after infection (Fig. 3). Furthermore, to confirm glial expression and morphology, we performed IF analysis. The results from IF demonstrated a significant increase in the expression of IBA-1-positive nuclei (*P* < 0.01) in the hippocampus 24 h after infection (Fig. 5) in the meningitis group. The expression of IBA-1 was increased in both the PFC (*P* < 0.01) and hippocampus (*P* < 0.01) in the 10-day meningitis group (Fig. 6). We also observed a significant increase in the expression of GFAP-positive cells in the PFC (*P* < 0.01) and hippocampus (*P* < 0.01) of the meningitis group, 24 h after infection, as compared to the levels in the control group (Fig. 7). Interestingly, persistent microglial activation was observed even 10 days after experimental meningitis. Similarly, the expression of GFAP was also elevated in both the PFC (*P* < 0.01) and hippocampus (*P* < 0.05) of the 10-day meningitis group compared to the levels of the controls (Fig. 8).

### Upregulation of TSPO expression and mitochondrial dysfunction after experimental meningitis

**Fig. 9:**
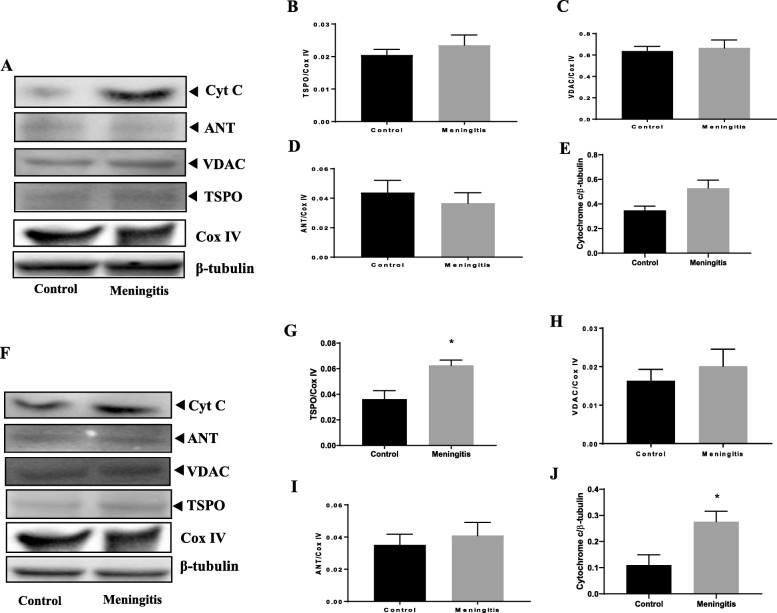
Upregulation of the expression of the neuroimmunomodulatory target TSPO 24 h after experimental meningitis induction in the PFC and hippocampus. Western blot analysis of the tissue protein levels of mitochondrial proteins. Quantification of the immunoblot data using the densitometric analysis of each protein was carried out using Image Lab™ software (Bio-Rad, CA, USA). Representative immunoblots and the quantification of TSPO, VDAC, ANT, cytochrome-*c* and loading controls, COX IV, and β-tubulin in PFC (**a**–**e**) and hippocampus (**f**–**j**). The values of all protein levels were normalized to COX IV or β-tubulin levels. The results are expressed as the mean ± SEM for *n* = 4–5 rats. *P < 0.05 compared to controls

**Fig. 10:**
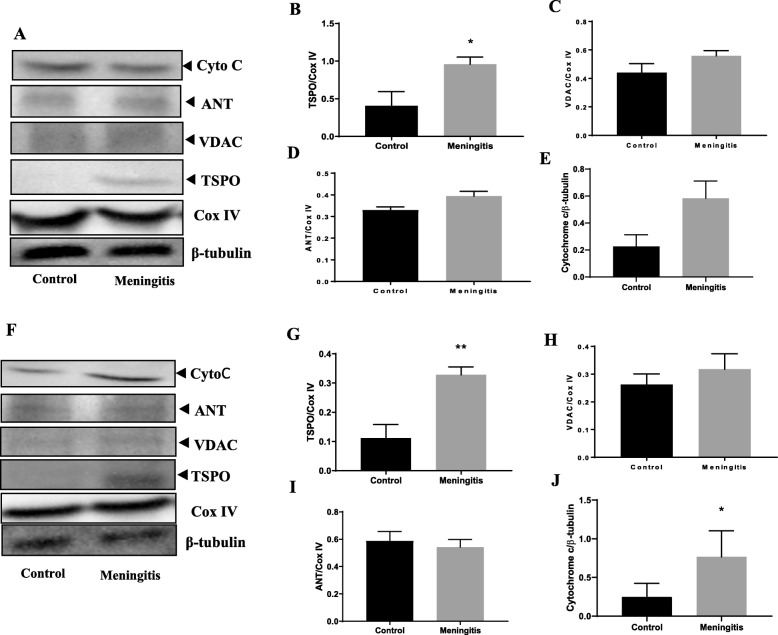
Upregulation of the expression of the neuroimmunomodulatory target TSPO 10 days after experimental meningitis induction in the PFC and hippocampus. Western blot analysis of the tissue protein levels of mitochondrial protein. Quantification of the immunoblot data using the densitometric analysis of each protein was carried out using Image Lab™ software (Bio-Rad, CA, USA). Representative immunoblots and the quantification of TSPO, VDAC, ANT, cytochrome-*c* and loading controls, COX IV, and β-tubulin in PFC (**a**–**e**) and hippocampus (**f**–**j**). The values of all protein levels were normalized to COX IV or β-tubulin levels. The results are expressed as the mean ± SEM for *n* = 4–5 rats. *P < 0.05 compared to controls

**Fig. 11:**
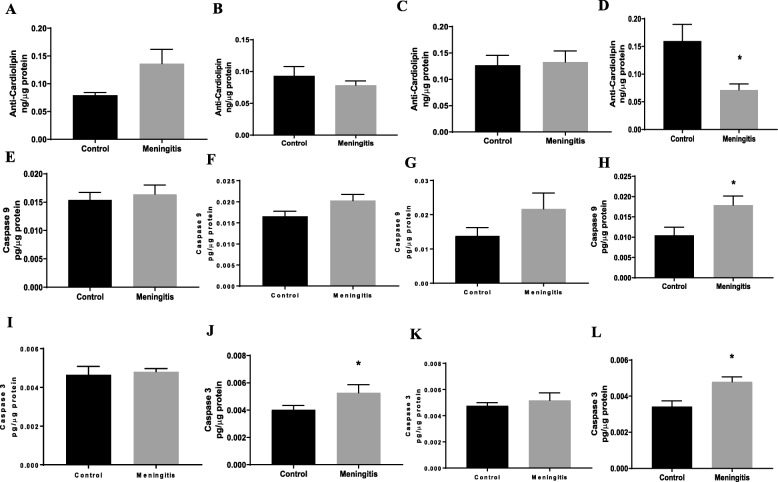
Decreased cardiolipin levels and elevated caspase levels after experimental meningitis. ELISA analysis for tissue protein levels of cardiolipin in experimental meningitis and control rats in the **a** PFC and **b** hippocampus 24 h after meningitis induction and in the **c** PFC and **d** hippocampus 10 days after induction. ELISA analysis for tissue protein levels of caspase-3 in experimental meningitis rats and control rats in the **e** PFC and **f** hippocampus 24 h after meningitis induction and in the **g** PFC and **h** hippocampus 10 days after induction. ELISA analysis for tissue protein levels of caspase-9 in experimental meningitis and control rats in the **i** PFC and **j** hippocampus 24 h after meningitis induction and in the **k** PFC and **l** hippocampus 10 days after induction. The results are expressed as the mean ± SEM for *n* = 5 rats. *P < 0.05 compared to controls

To understand the TSPO-mediated mitochondrial pathway, we measured the protein levels of the outer mitochondrial proteins TSPO and VDAC and inner mitochondrial protein ANT. Additionally, the levels of cytochrome-*c*, cardiolipin, caspase-3, and caspase-9 were evaluated in both the 24-h and 10-day groups. There was no significant change in the expression of TSPO, VDAC, and ANT at 24 h after meningitis induction, between the control and meningitis groups (Fig. 9). We observed elevated levels of hippocampal TSPO and cytochrome-*c* expression (*P* < 0.05) in the meningitis group, 24 h after meningitis induction, with no change in VDAC and ANT expression. Interestingly, the expression levels of TSPO in the meningitis group increased 10 days after infection in both the PFC (*P* < 0.05) and hippocampus (*P* < 0.01) (Fig. 10). These results are consistent with TSPO expression and reactive gliosis following CNS injury, in which glial cells upregulate TSPO [39]. In our study, TSPO expression was accompanied by the simultaneous upregulation of cytochrome-*c* levels (*P* < 0.05) in the 10-day group, with no significant change in VDAC and ANT levels. As we found an increase in cytochrome-*c* levels, we also analyzed the levels of cardiolipin using ELISA. We observed decreased hippocampal cardiolipin levels (*P* < 0.05) 10 days after meningitis infection (Fig. 11). To finish evaluating the mitochondrial pathway, we measured the levels of caspase-3 and caspase-9. Ten days after meningitis induction, the levels of both the cellular markers, caspase-3 and caspase-9, were increased (*P* < 0.05) in the hippocampus with no significant changes in the 24-h group except caspase-3 which was increased significantly (*P* < 0.05) in meningitis group hippocampus (Fig. 11).

### Cognitive decline assessed in meningitis survivor rats

#### Open-field test

**Fig. 12:**
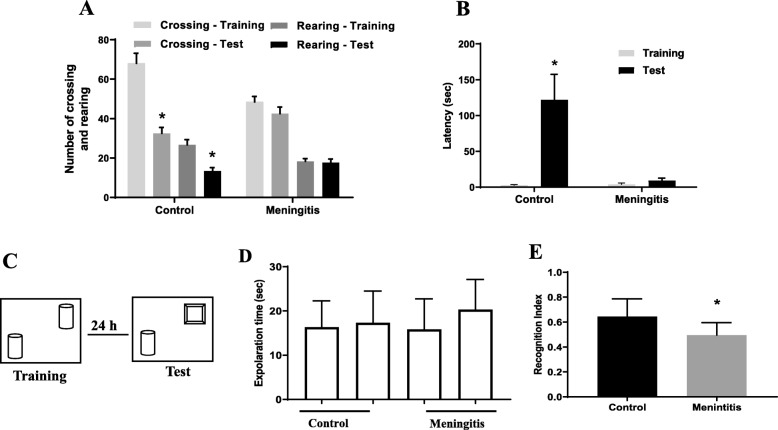
**a** Open-field test. **b** Step-down inhibitory avoidance task. **c** Schematic representation of the NOR test. **d** Locomotor activity. **e** Recognition index. The results are expressed as the mean ± SEM for *n* = 10 rats. *P < 0.05 compared to controls

The open-field test is used to evaluate habituation memory in rodents [40]. In the control group, we found significant differences between the training and test sessions, as measured by the number of crossings and rearings (*P* < 0.05). However, impairment in habituation memory was recognized in meningitis rats, with no difference in behavior between the training and test sessions (Fig. 12a).

#### Step-down inhibitory avoidance test

Aversive memory in rodents is evaluated by the step-down inhibitory avoidance test [41]. We found no difference in the latency to step-down in seconds between the training and test sessions in the experimental meningitis rats, demonstrating a decline in aversive memory. Conversely, in the control rats, there was a significant increase in the latency time, demonstrating aversive memory retention (*P* < 0.05; Fig. 12b).

#### Novel object recognition test

The novel object recognition task tests for recognition of familiar and novel objects and involves recollection and familiarity. The successful retrieval of the contextual details accompanying the learning episode denotes recollection. Familiarity involves identifying the item that was presented earlier [42]. We found that the rats subjected to experimental meningitis spent less time with the novel object. The meningitis group showed a significantly decreased recognition index (*P* < 0.05) compared to the control group. However, there were no significant differences in locomotor activity between the two groups (Fig. 12c–e).

## Discussion

Inflammation, the major immune response of CNS, is mediated by nonneuronal cells, which include the brain sentinels microglia and astrocytes. Although the initial neuroinflammatory response is essential to regulate the brain homeostasis, chronic or maladaptive inflammation leads to detrimental effects [43]. In line with this fact, we tested the experimental meningitis-induced inflammatory response in acute (24 h) and long-term (10 days) conditions. Twenty-four hours after meningitis induction, we observed increased in vivo microglial activation in the meningitis groups by TSPO-PET imaging. The trajectories of inflammation were further evidenced by increases in the levels of inflammatory cytokines, oxidative stress markers, and markers of glial activation in the 24-h group. As stated above, the initial increase in the inflammatory response after meningitis induction is imperative to eliminate the pathogen and to regulate brain equilibrium. However, it was intriguing to note that although the rats in the 10-day meningitis groups were free from infection, an increase in the inflammation was evidenced by a greater SUV of [^11^C]PBR28 in their whole brains. The subcellular changes that occur in the inflammatory marker TSPO levels upon the activation of microglia are not known. Upon activation, microglia change from a ramified morphology to an amoeboid morphology [44]. Transition to an amoeboid morphology may result in an increase in the mitochondrial population without a change in the amount of TSPO per mitochondrion. One possibility is that increased TSPO levels in activated microglia reflect an increase in the mitochondrial number. An increase in the amount of TSPO per mitochondrion without changes in the number of mitochondria might be the other possibility. Although it is not experimentally confirmed, the number of mitochondria, as well as the amount of TSPO per mitochondria, both likely increase with microglial activation, and these changes may have detrimental effects on the cells [7].

Consistent with our previous report, 10-day meningitis group rats demonstrated significant cognitive impairment in the battery of behavioral tests [17, 21]. The meningitis rats spent less time with novel objects, demonstrating recognition memory loss, as observed in the NOR test. In the step-down inhibitory avoidance task, we found no difference in the latency time between the test and training sessions, demonstrating a cognitive decline in meningitis rats. Furthermore, impairment in habituation memory was evidenced by the open-field task, in the meningitis group. Thus, we hypothesize that persistent microglial activation followed by TSPO-mediated neuroinflammation might play a role in the long-term cognitive decline exhibited in meningitis animals. TSPO is an evolutionarily conserved protein, and its primary function is to transport cholesterol to the inner mitochondrial membrane for the production of steroids. TSPO also plays a role in the regulation of many cellular processes, including inflammatory response, cytokine production, oxidative stress, mitochondrial homeostasis, and cell death [45, 46].

Knowing the role of TSPO in redox homeostasis, it is noteworthy to evaluate oxidative stress after meningitis [47]. We found exaggerated oxidative and nitrative stress markers, such as MDA, protein carbonyl, and nitrite/nitrate levels, in the PFC of the meningitis rats compared to those of the controls. A decrease in enzymatic defense, such as SOD and CAT levels, was observed in both the PFC and hippocampus of the meningitis rats. Srivastava et al. have reported clinical evidence for an increase in MDA, protein carbonyl, and nitrite levels and a decrease in enzymatic defense levels in the plasma and CSF of meningitis patients [48]. We also measured mitochondrial complex I, II, III, and IV activity in the PFC and hippocampus, and in the 24-h group, we found a decrease in complex III activity in the meningitis group compared to that in the control group (data not shown). Activated microglia and astrocytes in the CNS are the primary sources of cytokines during a reactive, inflammatory response [49]. The results from this study demonstrate a storm of inflammatory cytokines, such as IL1-α, IL-1β, IL-6, IL-18, TNF-α, and INF-γ, in the 24-h meningitis group. The persistent activation of microglia leads to increased inflammatory protein TNF-α and IL-4 levels, 10 days after meningitis induction. A recent study by Hennessy et al. has reported that elevated TNF-α levels have robust acute effects on brain function, including depression, delirium, and postoperative cognitive dysfunction [50]. Interestingly, other experimental evidence suggests that elevated IL-4 levels demonstrated hippocampal neuroinflammation and cognitive decline in a mouse model. IL-4 also plays a crucial role in hepatitis B vaccination-induced brain development and cognition [51].

In addition to the evaluation of microglial activation in vivo, we also confirmed the protein markers of glial cells in the PFC and hippocampus using conventional techniques. We found increased expression of microglial markers IBA-1 and CD11B in the meningitis rat hippocampus 10 days after meningitis induction. The strong association between the overexpression of glial proteins and cognitive impairment has been reported in different disease models [52, 53]. Because TSPO overexpression was also detected in reactive astrocytes [54], we determined GFAP protein expression, which was increased in both the 24-h and 10-day meningitis groups. Recently, a report from Clarke et al. revealed that pro-inflammatory microglia secrete IL-1α, TNF, and C1q, and these cytokines are sufficient to activate astrocytes, termed as A1 reactive astrocytes [55]. Furthermore, it was stated that A1-reactive astrocytes fail to perform normal functions and produce complement components that release toxic factors that, in turn, damage neurons and oligodendrocytes, thereby contributing to the cognitive decline in vulnerable brain regions in normal aging [55]. Interestingly, we found elevated TNF and C1q levels in the hippocampal region after 10 days of meningitis induction, suggesting the possibility of A1-reactive astrocytes induction after experimental meningitis. Although we found no difference in oligodendrocyte and neuronal protein expression, the cognitive decline recognized in meningitis survivors may be partially explained by persistent activation of microglia and astrocytes after experimental meningitis.

**Fig. 13:**
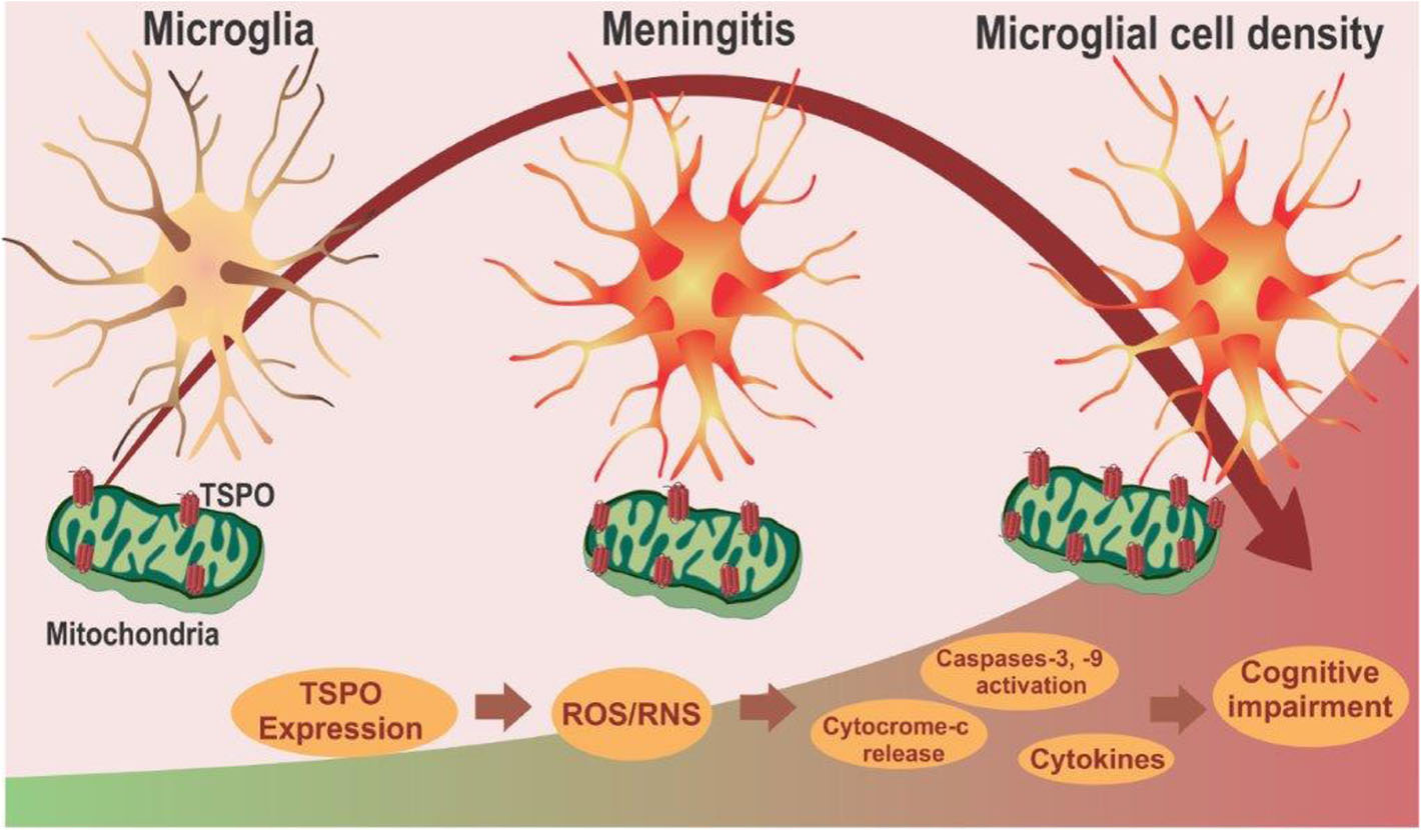
Experimental meningitis induced microglial activation. Induction of experimental meningitis increased the microglial activation, which in turn elevated the levels of TSPO expression. Further increase in oxidative and nitrosative stress and the interplay between activated glia-mediated immune responses through the TSPO mechanism underpins the cognitive decline observed in meningitis survivors

Recent emerging evidence strongly supports the use of TSPO as a neuroimmunomodulatory target to detect neuroinflammation in neurological and psychiatric disorders [56]. However, the role of TSPO in infection-induced inflammatory changes has not yet been explored. In our study, we confirmed the enhanced expression of TSPO in vivo, as well in the PFC and hippocampus of meningitis survivor rats. However, we found no differences in the levels of other mitochondrial proteins, namely, VDAC and ANT, but we documented increased expression of cytochrome-*c* in the hippocampus of meningitis rats at 10 days. It is important to note that cytochrome-*c* has been identified as a critical signaling molecule of apoptosis [57]. For the intrinsic apoptotic program, the redistribution of cardiolipin from the inner mitochondrial membrane to the outer mitochondrial membrane and the subsequent accumulation of cardiolipin oxidation products, catalyzed by cytochrome-*c*, are needed [58]. Oxidized cardiolipin causes the release of pro-apoptotic factors, including cytochrome-*c*, from mitochondria into the cytosol, which activates the caspases [58]. Cardiolipin content loss also contributes to the age-related phenotype generally associated with mitochondrial dysfunction and oxidative stress [59]. We found a significant increase in cytochrome-*c* levels 10 days after meningitis induction, with a simultaneous reduction in cardiolipin levels. The increased levels of cytochrome-*c* activated the caspase pathway by stimulating caspase-3 and caspase-9 levels, which were detected by ELISA. Our results are consistent with earlier reports by Bifrare et al. that confirmed the presence of apoptosis by documenting positive staining for activated, apoptosis-specific caspase-3 in an experimental meningitis model [60]. The current study demonstrated the presence of microglial activation in vivo by [^11^C]PBR28-PET imaging. Furthermore, the results also demonstrated the upregulation of TSPO, oxidative stress markers, and inflammatory mediator levels not only in the acute context but also in the long-term context (Fig. 13). Thus, in summary, it may be postulated that TSPO-dependent negative regulatory effects may play a role in the cognitive impairment affecting meningitis survivors.

## Conclusion

Based on our results, we posit that the dynamic interplay between the activated glia-mediated immune responses through the TSPO mechanism underpins the cognitive decline observed in meningitis survivors. Thus, [^11^C]PBR28-PET could be used as an imaging marker for the longitudinal monitoring of neuroinflammation in meningitis patients affected by progressive, long-term cognitive impairment. The limitations of the TSPO polymorphism can be bypassed by genotyping TSPO rs6971 and excluding individuals with the rare, low-affinity binding genotype. Additionally, these results also open a new avenue to target TSPO ligands in infection-induced long-term cognitive dysfunction.

## Data Availability

The datasets used and/or analyzed during the current study are available from the corresponding author on reasonable request.

## Abbreviations

ANT: Adenine nucleotide translocator
BCA: Bicinchoninic acid
BSA: Bovine serum albumin
CAT: Catalase
CFU: Colony-forming units
CNS: Central nervous system
CSF: Cerebrospinal fluid
FOV: Field-of-view
GFAP: Glial fibrillary acidic protein
i.c.: Intracisternal
IBA-1: Ionized calcium-binding adaptor molecule 1
IL: Interleukin
INF: Interferon
MDA: Malondialdehyde
MLEM: Maximum likelihood expectation maximization
MPO: Myeloperoxidase
MPTP: Mitochondrial permeability transition pore
PBR: Peripheral benzodiazepine receptor
PET: Positron emission tomography
PFC: Prefrontal cortex
PVDF: Polyvinylidenedifluoride
RT: Room temperature
SOD: Superoxide dismutase
SUV: Standard uptake volume
TBARS: Thiobarbituric acid reactive species
TNF: Tumor necrosis factor
TSPO: Translocator protein
VDAC: Voltage-dependent anion channel
